# Changes in inequality in utilization of preventive care services: evidence on China’s 2009 and 2015 health system reform

**DOI:** 10.1186/s12939-019-1078-z

**Published:** 2019-11-11

**Authors:** Yongjian Xu, Tao Zhang, Duolao Wang

**Affiliations:** 1 School of Public Policy and Administration, Xi’an Jiaotong University, Xi’an, Shaanxi China; 2 Department of Health Management, School of Medicine and Health Management, Tongji Medical College, Huazhong University of Science and Technology, Wuhan, Hubei China; 3 Department of Clinical Sciences, Liverpool School of Tropical Medicine, Liverpool, L3 5QA UK

**Keywords:** Preventive care, Inequality, Concentration index, Oaxaca decomposition, China

## Abstract

**Background:**

Ensuring equal access to preventive care has always been given a priority in health system throughout world. This study aimed to decompose inequality in utilization of preventive care services into its contributing factors and then explore its changes over the period of China’s 2009–2015 health system reform.

**Methods:**

The concentration index (CI) and decomposition of the CI was performed to capture income-related inequalities in preventive services utilization and identify contribution of various determinants to such inequality using data from China Health and Nutrition Survey. Then, changes in inequality from 2009 to 2015 were estimated using Oaxaca-type decomposition technique.

**Results:**

The CI for preventive services utilization dropped from 0.2240 in 2009 to 0.1825 in 2015. Residential location and household income made the biggest contributions to income-related inequalities in these two years. Oaxaca decomposition revealed changes in residential location, regions and medical insurance made positive contributions to decline in inequality. However, alternation in household income, age and medical services utilization pushed the equality toward deterioration.

**Conclusion:**

The pro-rich inequality in preventive healthcare services usage is evident in China despite a certain decline in such inequality during observation period. Policy actions on eliminating urban-rural and income disparity should be given the priority to equalize preventive healthcare.

## Introduction

Preventive healthcare is widely recognized as the most cost-effective services as it helps find and address health issues before people have any symptoms [1, 2]. For example, obtaining timely screening tests for certain cancers may mean diagnosis and treatment at early stage of the disease, thereby reducing patient’s disease economic risk, especially for the poor. Empirical evidences from previous studies revealed inputs to preventive health services can reduce treatment costs and save rescue costs significantly [2, 3]. Moreover, receiving regular preventive care was found to reduce premature mortality and improve quality of life [4, 5]. Therefore, uneven distribution of preventive healthcare services may result in growing inequalities in economic burden of disease and health between the poor and rich.

WHO has identified the equal access to prevention as a public health priority in the “Health for All” Agenda [6]. Simultaneously, ensuring even distribution of preventive care is also an important task to realize Sustainable Development Goal “promote well-being for all at all ages” announced by United Nations. Many countries have realized the importance of prevention and adopted targeted measures to ensure equitable accessibility of preventive healthcare [7, 8]. Although China has witnessed a rapid growth in economy over the past decades, population ageing poses a great challenge for entire society. In 2016, there were 230 million elderly people aged over 60, accounting for 16.7% of the total population, but the proportion of elderly people with chronic diseases was over 65% [9]. Therefore, preventive healthcare is especially important under such circumstances bin China. Indeed, a consensus of the need of shifting focus from disease-oriented to wellness and prevention was reached and “equalization of public health services” has become one of the major health-care policies in China [10, 11]. In order to ensure equal access to preventive care, Chinese governments made great efforts since the launching of the new round of healthcare system reform in 2009.

Universal health coverage in China is a nonnegligible accomplishment. Changes in health insurance coverage from approximately 56% in urban areas and around 21% in rural regions in 2003 to almost 95% in 2011 had improved access to medical services as well as preventive care [12, 13]. Additionally, National Essential Public Health Services Package (NEPHSP) was implemented to provide free public health services for urban and rural residents. For instance, vaccination for children aged 0–6 and health management for patients with chronic diseases were provided [14]. Moreover, Chinese government invested a lot to reduce financial barriers in preventive health services delivery. For example, funding subsidy for basic public health service increased from 15 Chinese yuan per capita in 2009 to 55 Chinese yuan per capita in 2018 [15].

Although these health reforms showed optimistic signs in the preventive health care, the challenges still persist. Unequal access to preventive health care is one of important issues to be identified and addressed. Liu and colleagues observed that there was a disparity between urban and rural in utilization of preventive care services after China’s health reform, and that income and education made a major contribution to this disparity [16]. Also, a study by Huang et al. indicated the level of preventive care usage was low among those who had low income, without a tertiary education and lived in a less affluent region [10]. In additional, a social gap in access to basic preventive care was found to exist before and after the 2009 health reform [13].

These studies provided some evidence on equalities in preventive health services, but gaps in these literatures need to fill. Firstly, there is scant literature examining socioeconomic-related inequalities in access to preventive healthcare using summary measures such as the concentration index (CI) and horizontal inequity index (HI). Secondly, little is known about changes in inequalities of preventive health services utilization and their associated contributor over the period of China’s health system reform. In such context, the present study aims to answer the following two questions: 1) Have inequality in utilization of preventive care services changed during the reform of China’s health care system? 2) What were the associated factors contributing to such change?

## Methods

### Data

In order to examine the change in inequality in preventive care usage over period of China’s health system reform, the data sets used in this study were from China Health and Nutrition Survey (CHNS) 2009 and 2015. CHNS is an ongoing nationally longitudinal study on nutrition, health insurance coverage, healthcare system, health behavior, social and economic transition in the Chinese society, and surveys began in 1989, with subsequent exams every 2 to 4 years, for a total of 10 rounds between 1989 and 2015 [10]. The survey areas originally covered nine provinces: Liaoning, Heilongjiang, Jiangsu, Shandong, Henan, Hubei, Hunan, Guangxi and Guizhou. In 2011 wave, Beijing, Shanghai and Chongqing were added. In each participating province or autonomous mega-city, a multistage random cluster process was used to select representative households and individuals [17]. All information was collected through face-to-face interviews. Details of the CHNS study protocol were published elsewhere [18, 19].

A total of 11,296 and 12,567 individuals participated in the survey in 2009 and 2015, respectively. After excluding data with key variables missing and logic error answers, 8574 in 2009 and 9514 in 2015 respondents were included for this study.

### Outcome variables

Preventive healthcare utilization was measured by asking respondents “During the past 4 weeks, did you receive any preventive health service?”. This service in the questionnaire contains health examinations, eye examinations, blood tests, blood-pressure screening, tumor screening, prenatal and postnatal examinations, and any other type of preventative examinations [10, 13, 16]. If the respondent used one of these preventive services, value was given 1. Otherwise the value was 0.

### Independent variables

Following by Andersen’s behavioral model [20], independent variables selected in the present study were divided into three categories: predisposing, enabling and need determinants. We classified gender, age and marital status as predisposing variables to reflect the individuals’ propensity to use health services. Enabling factors included education, employment status, medical insurance, annual household income per capita, residential location (urban/rural), region (east/central/west), and family size [13]. These variables represent financing and organizational conditions facilitating access to services. Need factors represent potential needs for health services. We used the questions “Have you ever been sick in the past 4 weeks” and “Have you ever received formal medical care in the past 4 weeks” to assess respondents’ needs (Table 1).

**Table 1:** Characteristics of study participants

	2009 (*n* = 8574)		2015 (*n* = 9514)	
	N/mean	%/S.D.	N/mean	%/S.D.
Preventive healthcare				
No	7976	93.0	8629	90.7
Yes	598	7.00	885	9.3
Gender				
Male	4158	48.5	4827	50.7
Female	4416	51.5	4687	49.3
Age	48.90	15.25	49.60	14.61
Marital status				
Unmarried	633	7.4	611	6.4
Married	7184	83.8	8387	88.2
Divorced / Widowed / Separated	757	8.8	516	5.4
Education				
Primary school and below	3400	39.5	1710	18.0
High school	4023	46.9	5224	54.9
Technical school	661	7.7	942	9.9
College and above	516	6.0	1638	17.2
Employment status				
No	3331	38.9	4492	47.2
Yes	5243	61.1	5022	52.8
Medical Insurance				
No	785	9.2	239	2.5
Yes	7789	90.8	9275	97.5
Household income (RMB)	11,064.35	1443.16	26,230.29	3961.71
Residential location				
Urban	2940	34.3	4007	42.1
Rural	5634	65.7	5507	57.9
Region				
East	3808	44.4	4957	52.1
Central	3736	43.6	3021	31.8
West	1030	12.0	1536	16.1
Family size	3.72	1.63	3.64	1.63
Disease status				
No	8006	93.4	8822	92.7
Yes	568	6.6	692	7.3
Medical services				
No	8464	98.7	9202	96.7
Yes	110	1.3	312	3.3

### Statistical analysis

#### Measuring inequality

CI, a widely accepted index, was used to depict inequalities in distribution of preventive healthcare. It quantified the degree of income-related inequality with a range from − 1 to 1. A negative value indicates a pro-poor effect with services being more concentrated on the poor, and vice versa. A zero value represents an absent of inequality [21]. The CI formula is as follows:

$$ \mathrm{C}=\frac{2}{\mu } COV\left(y,\gamma \right) $$

Where C was defined in terms of the covariance between the outcome variable (y) and the fractional ranks of household income (γ); μ is the mean of y.

#### Decomposing inequality

In order to analyze contribution of independent variables to the inequalities, we also followed the method proposed by Wagstaff et al. to decompose CI [22, 23]. Firstly, regression model on the outcome variable (y) was established:

$$ {y}_i={a}^m+{\sum}_k{\beta}_k^m{x}_{ki}+{\mu}_i $$

Where ${\beta}_k^m$ is the marginal effect (dy/dx) of each x; *μ*_*i*_ indicates the error term.

Then, the concentration index for y can be written as:

$$ \mathrm{C}={\sum}_k\left({\beta}_k\overline{x_k}/\mu \right){c}_k+{GC}_{\varepsilon }/\mu $$

Where *β*_*k*_ is the marginal effect of *x*_*k*_; $\overline{x_k}$ and *c*_*k*_ are the mean and the concentration index of *x*_*k*_; μ is the mean of y; *GC*_*ε*_ is the generalized concentration index for ε. This equation reveals the total concentration index consistent of two components: explained component and residual component. The first component contains two elements: 1) Elasticity ${\beta}_k\overline{x_k}/\mu$ as a unit-free measure of association that indicates the amount of change in dependent variable associated with one-unit change in explanatory variable. 2) *c*_*k*_ is the normalized CI of K variable. *GC*_*ε*_/*μ* represents the unexplained component which cannot be described by systematic variation in the determinants across economic groups.

#### Decomposing changes in inequality

At the final stage, we used Oaxaca-type decomposition to determine the extent to which change in inequality in preventive healthcare usage between 2009 and 2015 was owing to changes in inequality in the determinants [23–25]. The decomposition formula is as follows:

$$ \Delta  \mathrm{C}={\sum}_k{\eta}_{kt}\left({c}_{kt}-{c}_{kt-1}\right)+{\sum}_k{c}_{kt-1}\left({\eta}_{kt}-{\eta}_{kt-1}\right)+\Delta  \left(\raisebox{1ex}{${GC}_{\varepsilon t}$}\!\left/ \!\raisebox{-1ex}{${\mu}_t$}\right.\right) $$

Where *η*_*kt*_ and *η*_*kt* − 1_ represent the elasticities of explanatory variables in terms of preventive health services usage in 2009 and 2015, respectively. Accordingly, *c*_*kt*_ and *c*_*kt* − 1_ are the normalized CIs of explanatory variables in these two years, respectively. All data management and statistical analysis were performed on STATA 14.0.

## Results

### Characteristics of study participants

Table 1 provided descriptive statistics for key variables in 2009 and 2015. A slight rise in proportion of respondents who used preventive health services over past 4 weeks was observed during this period. Roughly equal proportion of men and women were presented in the sample in these two wave surveys. More than 80% participants got married. Mean age was between 48 and 50. Most of participants completed high school. More than half of respondents reported they were employed. The medical insurance coverage increased from 90.8% in 2009 to 97.5% in 2015. Due to a rapid growth in Chinese economy, annual household income per capita was doubled during this period. Most of people resided in rural and eastern provinces. Those people who reported suffering from illnesses or receiving formal medical care accounted for a small portion.

### Decomposition of inequality in utilization of preventive healthcare services

A positive CI value for preventive healthcare utilization was found in both 2009 (CI = 0.2240) and 2015 (CI = 0.1825), indicating a pro-rich effect (*p* < 0.05). In other word, the rich people were more likely to use preventive health services frequently than their poor counterparts.

**Fig. 1:**
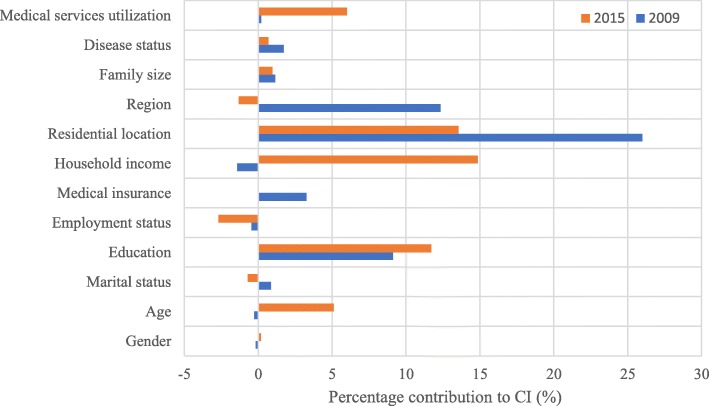
percentage contribution of determinants to CI of preventive health services utilization

The results from decomposition of inequalities in access to preventive healthcare in 2009 and 2015 were reported in Table 2. Overall, those residing in rural (25.99% in 2009; 13.55% in 2015) made a major contribution to the pro-rich distribution of preventive care in two rounds of investigation, despite a decline appeared in the second wave investigation (Fig. 1). It means that rural residents are less likely to use preventive health services. Additionally, the educational level was also an important contributor for such inequality, especially in respondents completed technical school or college.

**Table 2:** Decomposition of concentration index of preventive health services utilization

	2009 (*n* = 8574)				2015 (*n* = 9514)			
	C_k_	Elasticity	Absolute contribution	Percentage Contribution (%)	C_k_	Elasticity	Absolute Contribution	Percentage Contribution(%)
Gender								
Male	Ref.				Ref.			
Female	−0.0051	0.0761	−0.0004	−0.17	0.0023	0.1397	0.0003	0.18
Age	0.0027	−0.2342*	− 0.0006	− 0.28	0.0155	0.6021*	0.0093	5.11
Marital status								
Unmarried	Ref.				Ref.			
Married	0.0122	0.1417*	0.0017	0.77	−0.0014	0.2067*	− 0.0003	− 0.16
Divorced / Widowed / Separated	− 0.1261	− 0.0017	0.0002	0.10	− 0.0359	0.0279	− 0.0010	− 0.55
Education								
Primary school and below	Ref.				Ref.			
High school	0.0179	0.1037*	0.0019	0.83	− 0.0802	− 0.0177	0.0014	0.78
Technical school	0.3310	0.0281*	0.0093	4.15	0.1770	−0.0010	− 0.0002	− 0.10
College and above	0.5085	0.0182*	0.0093	4.13	0.3737	0.0543*	.0203	11.12
Employment status								
No	Ref.				Ref.			
Yes	0.0224	−0.0469	−0.0011	− 0.47	0.0592	− 0.0828	− 0.0049	−2.69
Medical Insurance								
No	Ref.				Ref.			
Yes	0.0103	0.7096*	0.0073	3.26	0.0012	0.0471*	0.0001	0.03
Household income (RMB)	0.4863	−0.0066*	− 0.0032	−1.43	0.5059	0.0536*	0.0271	14.86
Residential location								
Urban	Ref.				Ref.			
Rural	−0.0379	− 1.5362*	0.0582	25.99	−0.0483	− 0.5121*	0.0247	13.55
Region								
East	Ref.				Ref.			
Central	−0.0393	−0.1955*	0.0077	3.43	−0.1192	−0.0463*	0.0055	3.02
West	−0.0732	−0.2723*	0.0199	8.90	−0.2911	0.0273	−0.0079	−4.35
Family size	−0.2207	−0.0116	0.0026	1.14	−0.0835	−0.0213	0.0018	0.97
Disease status								
No	Ref.				Ref.			
Yes	0.0760	0.0508	0.0039	1.72	0.0537	0.0236	.0013	0.69
Medical services								
No	Ref.				Ref.			
Yes	0.0533	0.0086	0.0005	0.20	0.2477	0.0443	.0110	6.01

* *p* < 0.05

Compared with respondents from eastern region, those who were from central and western provinces had a smaller probability to access to preventive care in 2009. However, this disparity was narrowed in 2015. Notably, percentage contribution from annual household income per capita to the uneven distribution of preventive healthcare raised from − 1.43 to 14.86% during the observation period. It indicates that the change in household income worsened such pro-rich inequality considerably.

Except the above, other factors, such as age (5.11%), medical services (6.01%) and employment status (− 2.69%), also showed a substantial contribution to the observed pro-rich inequality in 2015, even though contribution importance of these variables were smaller in 2009. Furthermore, participants covered by medical insurance contributed 3.26% to the increase in such inequality in 2009, but decreased to 0.03% in 2015. This change implied that the universal health coverage made a difference in reducing inequality in preventive healthcare. For remaining variables such as gender, marital status, the percentage contribution was very small during these two periods.

### Decomposing changes in inequality in utilization of preventive healthcare services between 2009 and 2015

**Fig. 2:**
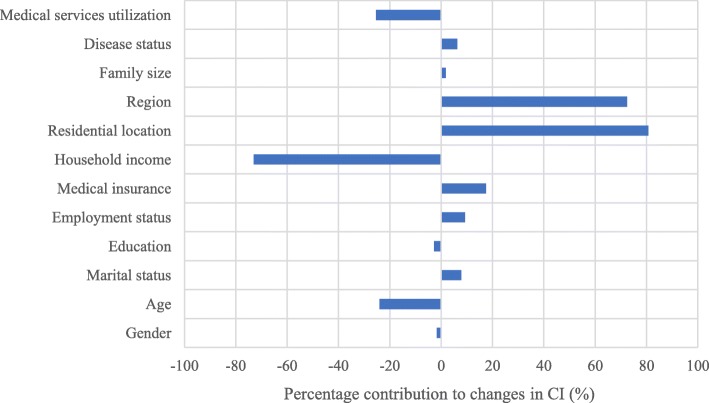
contribution from changes in independent variables to changes in CI of preventive survives utilization

As shown above, CI of preventive services utilization reduced by 0.0415 (18.5%) from 2009 to 2015. Then, this reduction was decomposed to seek contributing factors following by Oaxaca-type decomposition. The results were presented in Table 3 and Fig. 2.

**Table 3:** Oaxaca-type decomposition for changes in inequality in preventive health services utilization, 2009–2015

	Δc*η_kt_	Δη*c_kt-1_	Total	Percentage (%)
Gender				
Male	Ref.			
Female	0.0010	−0.0003	0.0007	−1.71
Age	0.0077	0.0023	0.0100	−24.01
Marital status				
Unmarried	Ref.			
Married	−0.0028	0.0008	−0.0020	4.86
Divorced / Widowed / Separated	0.0025	−0.0037	−0.0012	2.93
Education				
Primary school and below	Ref.			
High school	0.0017	−0.0022	−0.0004	1.05
Technical school	0.0002	−0.0096	−0.0095	22.84
College and above	−0.0073	0.0184	0.0110	−26.60
Employment status				
No	Ref.			
Yes	−0.0030	−0.0008	−0.0039	9.28
Medical Insurance				
No	Ref.			
Yes	−0.0004	−0.0068	−0.0073	17.48
Household income (RMB)	0.0011	0.0293	0.0303	−73.07
Residential location				
Urban	Ref.			
Rural	0.0053	−0.0388	−0.0335	80.69
Region				
East	Ref.			
Central	0.0037	−0.0059	−0.0022	5.21
West	−0.0059	−0.0219	− 0.0279	67.18
Family size	−0.0029	0.0021	−0.0008	1.88
Disease status				
No	Ref.			
Yes	−0.0005	−0.0021	−0.0026	6.25
Medical services				
No	Ref.			
Yes	0.0086	0.0019	0.0105	−25.34

Overall, changed CI and elasticities of all independent variables contributed differently to the reduction in inequalities in preventive services utilization. Region (72.39%) and residential location (80.69%) accounted for the largest contributions to the observed decrease in inequality, which mainly due to changes of these two variables in elasticity rather than the unequal distribution. It can be inferred that effects of urban-rural and regional disparities on inequality of preventive care decreased significantly. Additionally, changes in medical insurance coverage (17.48%), employment status (9.28%) and marital status (7.79%) can explain the reduction of CI to some extent.

However, annual household income per capita (− 73.07%) was found to become the biggest contributor for the increase in CI of preventive care. In other word, a widening income gap further worsened uneven distribution of preventive services. Also, changes in age (− 24.01%) and medical services utilization (− 25.34%) pushed such inequality into the deterioration.

Interestingly, we observed that contribution from the change in technical school and college was opposite, which resulted in total effect for education as an offset.

## Discussion

This study sheds some insights into the changes in income-related inequalities in preventive care services usage from 2009 to 2015 in China. The main findings were as following: 1) the pro-rich inequality in preventive health services utilization existed in both periods, but such inequality decreased by 18.5% over time. 2) The change in inequality attributed to the alteration in the interaction among the related determinants.

Overall, the finding showed an encouraging sign due to the decline in inequality of preventive health services utilization despite a pro-rich inequality still persisted. In recent years, Chinese governments made great efforts to equalize basic public health services after the new round of health care system reform [14, 26, 27]. The establishment of a three-level preventive healthcare service network in rural areas and the provision of physical examinations for the elderly free of charge are such examples [28]. Naturally, these initiatives are supposed to help reduce uneven distribution of preventive care.

Similar to other studies in the field of medical services [29, 30], the significant unequal utilization of preventive care services between rural and urban was observed though decomposition of CI, whereas the substantial decline in such inequality in 2015 was found. Also, region was seen as a vital contributor of the observed inequality. For a long time, the China’s rural-urban and regional disparity in economic level caused many problems in the field of healthcare, such as distribution of health services [31, 32]. According to the National Health Statistics Yearbook in 2016, the number of health technician per 1000 persons reached 11.1 in the eastern urban areas, while 3.7 in western rural areas [33]. Predictably, such serious shortage of health workforce in rural and western regions limits preventive health services delivery and usage largely.

Additionally, household income was identified as the biggest contributor to unequal access to preventive healthcare in 2015. Actually, evidences from previous studies proved income was associated with health services utilization including preventive health services because a high income means a high payment capacity for healthcare [10, 11, 34]. In line with another study [13], educational level also can help to explain the uneven distribution of preventive healthcare over this period. Generally, those with high educational attainment have more knowledge about disease prevention and a higher level of awareness for the needs of preventive care [35], thereby driving preventive services utilization.

Oaxaca decomposition revealed that the reduction in inequality arose from the alteration in the interaction among the related determinants. Changes in residential location, regions and medical insurance were observed to have made a major contribution to reduction of inequality. This finding mainly linked to fact that Chinese governments strived to establish the public health system covering rural and urban residents and universal health insurance system since 2009 health system reform. For example, expansion of basic medical insurance coverage reduced a financial burden in seeking health services, especially for the poor [36]. Additionally, other studies also elaborated that plenty of funds as well as health resources were inputted into rural and undeveloped areas, which helped bridge urban-rural and regional disparities in distribution of preventive care [30, 31].

However, we observed that alterations in household income, age and medical services use pushed the inequality in preventive health services usage towards deterioration. A possible explanation is related to the population ageing and widening income gap in China. Previous studies showed low-income families tended to spend a large proportion of disposable income on basic living needs rarely involved in preventive health services [37]. Accordingly, inequality of income in China was continuing to increase over the past few decades [38], ultimately expanding the gap in purchasing healthcare services between the rich and poor. Moreover, the rapid growth in older population in China resulted in an increased chronic illness and disability, and accordingly higher needs for healthcare [39]. Therefore, more preventive care services were biased toward those people.

Also, the results indicated that changes in those used medical services over past 4 weeks made a negative contribution to the reduction in such inequality. It was presumed that this group of people seemed to have high needs for health services. Simultaneously, the evidence that improved supply capacity of public health services over such period reduced barrier to seek preventive care [26, 28] can help to explain why those people used more preventive services in comparison to the past.

Several limitations in this study should be mentioned. Firstly, using single one variable is limited to assessed preventive health services utilization due to non-availability of other variables in CHNS. Secondly, independent variables used in decomposition of inequality mainly contained characteristics of respondents, rarely involved in supply-side factors affecting preventive services use, such as the distribution density of health workers, price of preventive healthcare. Thirdly, only 7 years were observed since 2009 because CHNS data is currently updated to 2015. Therefore, further study should be focused on changes in inequality over a longer period after China’s health system reform if data is available. Finally, causal interpretations should be made with caution since data were drawn from a cross-sectional study.

In spite of these shortcomings, we have extended current research using a national representative sample and a frequently used methodology to measure inequality in preventive services utilization. Additionally, this study also provided a deep understanding on the change in uneven distribution of preventive care during the new round of health care reform in China.

## Conclusion

Overall, preventive healthcare is in favor of the rich in China in spite of a certain degree of decline in such inequality from 2009 to 2015. The Oaxaca decomposition analysis suggested that the reduction in pro-rich inequality mainly attributed to the narrowed urban-rural and regional disparities in terms of healthcare delivery. However, a widening income gap further worsened inequality in preventive healthcare during such period. Policies should still promote balanced development among different regions, and emphasize on eliminating the gaps between rich and poor. In addition, universal health insurance system also should be designed to cover basic preventive health services for all people.

## Data Availability

The data used in this study can be found at https://www.cpc.unc.edu/projects/china.

## Abbreviations

CHNS: China Health and Nutrition Survey
CI: Concentration index
NEPHSP: National Essential Public Health Services Package
